# Listeriosis in a Metropolitan Hospital: Is Targeted Therapy a Risk Factor for Infection?

**DOI:** 10.3389/fmed.2022.888038

**Published:** 2022-04-29

**Authors:** Fanfan Xing, Simon K. F. Lo, Susanna K. P. Lau, Patrick C. Y. Woo

**Affiliations:** ^1^Department of Clinical Microbiology and Infection Control, The University of Hong Kong - Shenzhen Hospital, Shenzhen, China; ^2^Department of Microbiology, Li Ka Shing Faculty of Medicine, The University of Hong Kong, Hong Kong, Hong Kong SAR, China

**Keywords:** *Listeria monocytogenes*, opportunistic infection, targeted therapy, EGFR, HER2

## Abstract

Targeted therapies are widely used for treatment of autoimmune diseases as well as solid organ and hematological malignancies. Various opportunistic infections have been described in patients on targeted therapies. Although case reports or a few case series of listeriosis have been reported to be associated with targeted therapy, most of the cases were related to anti-tumor necrosis factor-α monoclonal antibody. In this study, we describe the epidemiological and clinical profiles of listeriosis in a tertiary hospital in Shenzhen, a Southern Chinese metropolitan city in China. During the 9-year-and-6-month study period, a total of five cases of listeriosis were recorded and all of them had *Listeria monocytogenes* bacteremia. All five patients had predisposing factors, including corticosteroid (*n* = 3), targeted therapy (*n* = 2), pregnancy (*n* = 2) and anti-interferon gamma autoantibody (*n* = 1). The two patients who had targeted therapy during their course of cancer treatment received inhibitors of the epidermal growth factor receptor (EGFR)/human epidermal growth factor receptor 2 (HER2) pathway. The first one was a 52-year-old woman with metastatic adenocarcinoma of the lung. She was given gefitinib (EGFR tyrosine kinase inhibitor), osimertinib (third-generation EGFR tyrosine kinase inhibitor) and afatinib (tyrosine kinase inhibitor that can bind to EGFR, HER2 and HER4). The second one was a 40-year-old woman with carcinoma of the breast with brain metastasis. She was given trastuzumab (anti-HER2 monoclonal antibody) and lapatinib (dual tyrosine kinase inhibitor of the EGFR/HER2 pathway). These two patients represent the second and third reports of listeria infections associated with EGFR/HER2 pathway inhibitors in the literature. Targeted therapy is an important predisposing factor for listeriosis. Listeria infection is an important differential diagnosis in patients on targeted therapy who present with sepsis and/or central nervous system infection, and the use of antibiotic regimens that cover listeria is crucial for empirical treatment. Avoidance of high-risk food items in these patients is important for the prevention of listeriosis.

## Introduction

Listeriosis is a food-borne disease caused by *Listeria monocytogenes* or *Listeria ivanovii*, often due to ingestion of unpasteurized milk and dairy products as well as contaminated undercooked meat, ice-cream, salads, and other ready-to-eat items. However, in a significant proportion of patients, the contaminated food source may not be obvious. In immunocompetent hosts, listeriosis usually manifests as a self-limiting febrile gastroenteritis. Nevertheless, in patients of extreme age, pregnant women, and other immunodeficiency states, it may lead to systemic sepsis with bacteremia, disseminated abscesses in multiple organs especially liver and spleen, and most severely central nervous system infections including meningitis, rhombencephalitis, cerebritis and brain abscess. As *L. monocytogenes* and *L. ivanovii* are intracellular pathogens, the immunocompromised patients with listeriosis are often those with cell-mediated immunity defects, such as patients receiving corticosteroids, transplant recipients, and HIV carriers.

Targeted therapies are widely used for treatment of various kinds of autoimmune diseases as well as solid organ and hematological malignancies. Since the targets of such therapies are usually major players of immunological pathways, they are often associated with infectious diseases that require the particular type of immune response for control. Various opportunistic infections have been described to be associated with different targeted therapies, the commonest reported ones being tuberculosis and invasive fungal infections (1–4). Although case reports or a few case series of listeriosis have been reported to be associated with targeted therapy, most of the cases were related to anti-tumor necrosis factor-α (anti-TNFα) monoclonal antibody (5–36). Recently, we described the first report of *L. monocytogenes* bacteremia and severe sepsis complicating carfilzomib (proteosome inhibitor) and daratumumab (anti-CD38 monoclonal antibody) treatment for multiple myeloma (37). In this study, we describe the epidemiology and clinical course of listeria infections in a tertiary hospital in Shenzhen, a Southern Chinese metropolitan city with a large immigrant population from other parts of China. The importance of targeted therapy as a risk factor of listeriosis is also discussed.

## Materials and Methods

### Patients

This was a retrospective study conducted over a nine-year-and-six-month period (1 July 2012 to 31 December 2021) in The University of Hong Kong - Shenzhen Hospital. Many newer medications, which have not been registered or come into the market in mainland China, are available in Shenzhen Hospital of the University of Hong Kong. The clinical details, laboratory data and radiological findings of all patients with listeriosis were retrieved from the hospital electronic record system and were analyzed. Ethics approval for this study was provided by the Institutional Review Board of The University of Hong Kong - Shenzhen Hospital ([2022]041).

### Microbiological Methods

Clinical specimens were collected and handled according to standard protocols, and all suspect colonies were identified by standard conventional biochemical method (38). The BacT/ALERT 3D(240) blood culture system (Becton Dickinson, Maryland, USA) was used. *L. monocytogenes* was suspected when facultative anaerobic Gram-positive non-sporulating rods with tumbling motility at room temperature were isolated on sheep blood agar. All identifications were confirmed by matrix-assisted laser desorption ionization time-of-flight mass spectrometry by the direct transfer method using the MALDI–TOF MS spectrometer (Bruker Daltonik) and spectra analyzed with IVD MALDI Biotyper 2.3 and reference library DB-9607 (Bruker Daltonik) (39). *In vitro* susceptibility in terms of minimal inhibitory concentrations of penicillin on the *L. monocytogenes* strains was performed using Epsilometer-test (Liofilchem^®^), with quality control strain *Streptococcus pneumoniae* ATCC49619 as the reference strain.

## Results

### Epidemiology of Lsteriosis

During the study period, a total of five cases of listeriosis were recorded (Table 1). One patient was a male and the other four were females. Median age was 40 years (range: 27–59 years). All five patients had predisposing factors for listeriosis, including corticosteroid (*n* = 3), targeted therapy (*n* = 2), pregnancy (*n* = 2) and anti-interferon gamma autoantibody (*n* = 1). Two patients had consumed suspected food items which may be the source of the infection. Case 3 had eaten leftover meat and vegetable stored in the refrigerator for a few days, whereas case 4 had bird's nest which had also been stored in the refrigerator for 6 days.

**Table 1:** Patients with listeriosis in the present cohort.

**Case no**.	**Sex/Age**	**Underlying disease(s)**	**Immunosuppressive treatment**	**Suspected contaminated food**	**Clinical samples where listeria was detected**	**Antibiotics**	**Outcome**
1	F/52	Adenocarcinoma of lung with metastasis	Pemetrexed, cisplantin, dexamethasone, gefitinib, osimertinib, afatinib, radiotherapy	None	Blood	None	Succumbed
2	F/40	Breast carcinoma with metastasis	Paclitaxel, dexamethasone, trastuzumab, lapatinib	None	Blood	Ampicillin + gentamicin	Recovered
3	F/27	Systemic lupus erythematosus, autoimmune hepatitis, pregnancy	Prednisone, hydroxychloroquine	Leftover meat and vegetable stored in refrigerator	Blood	Ampicillin	Recovered, abortion
4	F/40	Sjögren's syndrome, pregnancy	Prednisone, hydroxychloroquine	Bird's nest stored in refrigerator	Blood, placenta	Ampicillin	Recovered, abortion
5 (40)	M/59	Anti-interferon gamma autoantibody	None	None	Blood, CSF	Ampicillin	Recovered

*CSF, cerebral spinal fluid*.

### Clinical Course and Outcome

Case 1 was diagnosed to have metastatic adenocarcinoma of the lung in 2014. She was given gefitinib from November 2014 to October 2015, osimertinib from October 2015 to October 2016, and pemetrexed and cisplatinum from November 2016 to April 2017. Due to severe headache, she was admitted in June 2017 for brain radiotherapy. On admission, the total white cell count and neutrophils were within normal range, but lymphocytes were 0.87 × 10^9^/L. Liver and renal function tests were normal. C-reaction protein was <5 mg/L. After admission, dexamethasone and whole brain radiotherapy were commenced. 25 days after admission, she developed urinary incontinence, bilateral lower limb weakness and coma. 2 days later, she started to have fever but there were no localizing signs. The total white cell count and neutrophils were elevated to 10.31 × 10^9^/L and 9.33 × 10^9^/L, respectively, and lymphocytes were decreased to 0.51 × 10^9^/L. Liver and renal function tests remained normal. C-reaction protein was elevated to 67.9 mg/L. Then, Afatinib was commenced. On the next day, blood culture was collected but unfortunately the patient succumbed. 2 days later, blood culture yielded a Gram-positive bacillus, which was subsequently identified as *L. monocytogenes*.

Case 2 was diagnosed to have carcinoma of the breast with brain metastasis in April 2018. She was given paclitaxel, cisplatinum, trastuzumab and lapatinib from April to October 2018. Due to right upper limb weakness, oral dexamethasone was started in October 2018. 1 week after the commencement of dexamethasone, she was admitted for whole brain radiotherapy. On admission, she had fever without localizing symptoms or signs. The total white cell count and neutrophils were normal, but lymphocytes and platelet were 0.43 × 10^9^/L and 56 × 10^9^/L, respectively. Liver function test was mildly deranged. Renal function test was normal. Blood culture was performed and a Gram-positive bacillus was isolated from blood culture after 17 hours of incubation. The bacterium was subsequently identified as *L. monocytogenes*. The minimum inhibitory concentration of penicillin was 0.25 μg/mL. High dose intravenous ampicillin and gentamicin were commenced. Magnetic resonance imaging of the brain showed multiple metastatic lesions in the cerebrum, cerebellum and brainstem. Lumbar puncture was not performed because of the low platelet count. She became afebrile 3 days later and the antibiotics were kept for a total of 21 days.

Case 3 was diagnosed to have systemic lupus erythematosus and autoimmune hepatitis in 2015 and she had been put on prednisone and hydroxychloroquine since then. At her 19th week of gestation, she was admitted in March 2019 because of fever, chills and headache for 5 days. There were no localizing features. The total white cell count and neutrophils were elevated to 21.2 × 10^9^/L and 17.8 × 10^9^/L, respectively. Liver and renal function tests were normal. C-reactive protein was elevated at 40 mg/L. Blood culture was performed and empirical intravenous meropenem was commenced. Spontaneous abortion developed 2 days after admission. On the same day, blood culture yielded a Gram-positive bacillus, which was subsequently identified as *L. monocytogenes*. Contrast-enhanced computed tomography of the brain did not reveal any abnormality. Lumbar puncture was refused by the patient. Then, meropenem was stopped and intravenous ampicillin was commenced. She became afebrile 3 days later and antibiotics were kept for a total of 17 days.

Case 4 was diagnosed to have Sjögren's syndrome in November 2018 and she had been put on prednisone and hydroxychloroquine since then. Hydroxychloroquine was also added in March 2019. At her 29th week of gestation, she was admitted in August 2019 because of fever, chills, rigor and absence of fetal movement for 3 days. There were no localizing features. The total white cell count and neutrophils were elevated to 17.0 × 10^9^/L and 13.7 × 10^9^/L, respectively. Liver and renal function tests were normal. C-reactive protein levels were high (93.6 mg/L). Blood culture was performed and empirical intravenous meropenem was commenced and labor was induced. After 24 h of incubation, blood culture yielded a Gram-positive bacillus, which was subsequently identified as *L. monocytogenes*. Meropenem was stopped and intravenous ampicillin was commenced. Examination of the cerebrospinal fluid revealed no abnormality. She became afebrile 2 days later and antibiotics were kept for a total of 15 days. Histopathological examination of the placenta showed severe acute chorioamnionitis involving the extraplacental membranes, acute villitis, intervillous abscess formation and retroplacental hematoma. *L. monocytogenes* was also isolated from the placental tissue.

The detailed clinical course of Case 5 has been reported elsewhere (40). Briefly, case 5 had a history of *Talaromyces marneffei* infection in March 2019 and was diagnosed to have anti-interferon gamma autoantibodies in May 2021. He was admitted in June 2021 because of acute onset of fever, severe headache and malaise. The total white cell count and neutrophils were elevated to 24.5 × 10^9^/L and 21.2 × 10^9^/L, respectively. Liver and renal function tests were mildly deranged. C-reactive protein was elevated at 282.4 mg/L. Blood culture was performed. Empirical intravenous piperacillin-tazobactam and voriconazole were commenced. A Gram-positive bacillus was isolated from blood culture after 21 h of incubation. The bacterium was subsequently identified as *L. monocytogenes*. Examination of the cerebrospinal fluid showed a cell count of 387 × 10^6^/L, with 8% neutrophils, 10% lymphocytes and 82% monocytes, glucose of 2.8 mmol/L and protein of 373 mg/L. Bacterial culture was negative but next-generation sequencing revealed *L. monocytogenes* DNA. Piperacillin-tazobactam was stopped and intravenous ampicillin was commenced. Subsequently, the patient was also diagnosed to have disseminated *Mycobacterium kansasii* infection and was treated accordingly.

## Discussion

During the last decade or so, there has been a marked increase in the success in the elucidation of the molecular mechanisms behind primary immunodeficiency syndromes that result in opportunistic infections. Some of these syndromes include anti-interferon gamma autoantibody (an immunological defect typically associated with infections caused by intracellular pathogens) and mutations in genes that encode key players of various cellular and immunological pathways (e.g., *STAT3, TSC2*) (41). In the same period of time, the surge in the use of targeted therapies, such as kinase inhibitors and antibodies against various targets in the cellular and immunological pathways, has led to secondary immunodeficiency; which in turn has resulted in opportunistic infections (2). In the present cohort, all the five cases with listeriosis in our hospital had predisposing factors, some of the well-known ones being pregnancy (case 3 and 4) and corticosteroid therapy (case 1, 2 and 3). On the other hand, it is of note that 60% of the cases had either the above-mentioned primary immunodeficiency syndrome (case 5) or secondary immunodeficiency induced by targeted therapy (case 1 and 2). In fact, the case of listeria bacteremic meningitis (case 5) was the first report of listeriosis in a patient with anti-interferon gamma autoantibody in the literature (40). For the two patients who had targeted therapy (case 1 and 2), both of them received inhibitors of the EGFR/HER2 pathway during their course of lung and breast cancer therapy, respectively. Gefitinib and osimertinib are EGFR tyrosine kinase inhibitors, lapatinib is a dual tyrosine kinase inhibitor on the EGFR/HER2 pathway, afatinib is a tyrosine kinase inhibitor that can bind to EGFR, HER2 and HER4, and trastuzumab is an anti-HER2 monoclonal antibody. Although it has been shown in various studies and meta-analysis that inhibitors of the EGFR/HER2 pathway were associated with higher risks and severity of infections (42–44), the relative contribution of them is often difficult to quantify, as these patients are usually prescribed with other medications, such as corticosteroids, with immunosuppressive effects. In fact, both case 1 and 2 have also been given corticosteroid and chemotherapy for their underlying malignancies in addition to the EGFR/HER2 pathway inhibitors.

**Figure 1:**
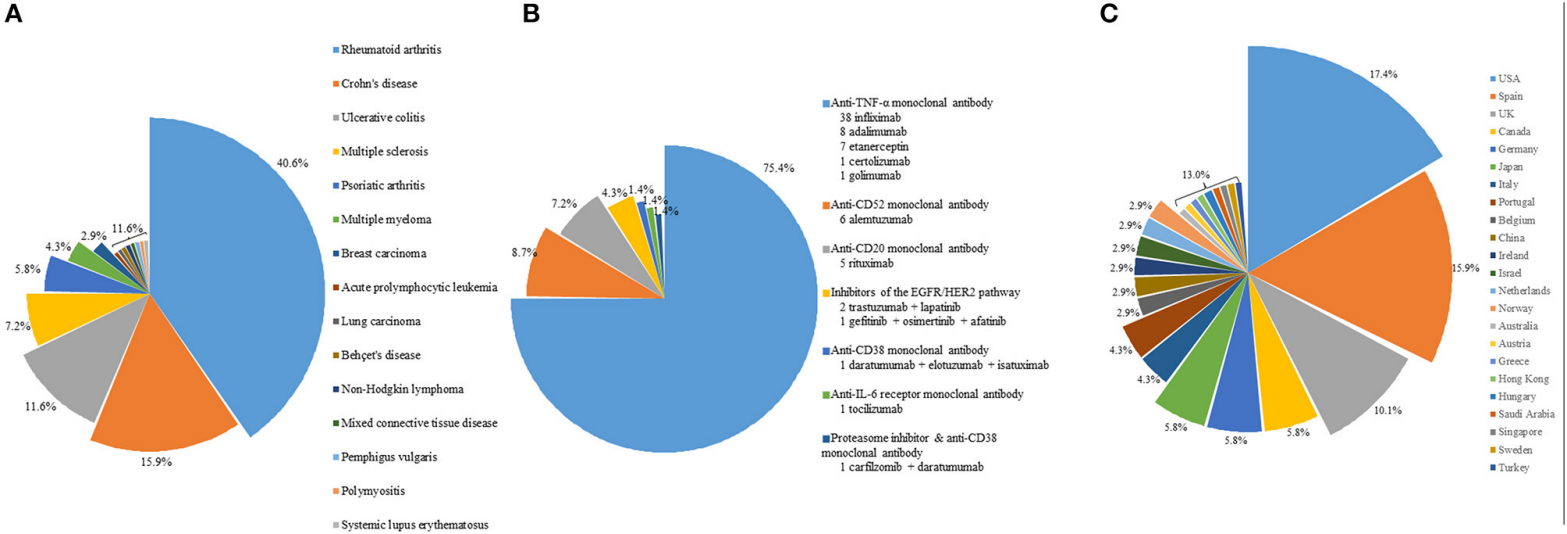
**(A)** Distribution of underlying diseases in patients on targeted therapy with listeriosis. **(B)** Distribution of the type of targeted therapy in these patients. **(C)** Distribution of these patients with listeriosis in different countries or regions.

**Figure 2:**
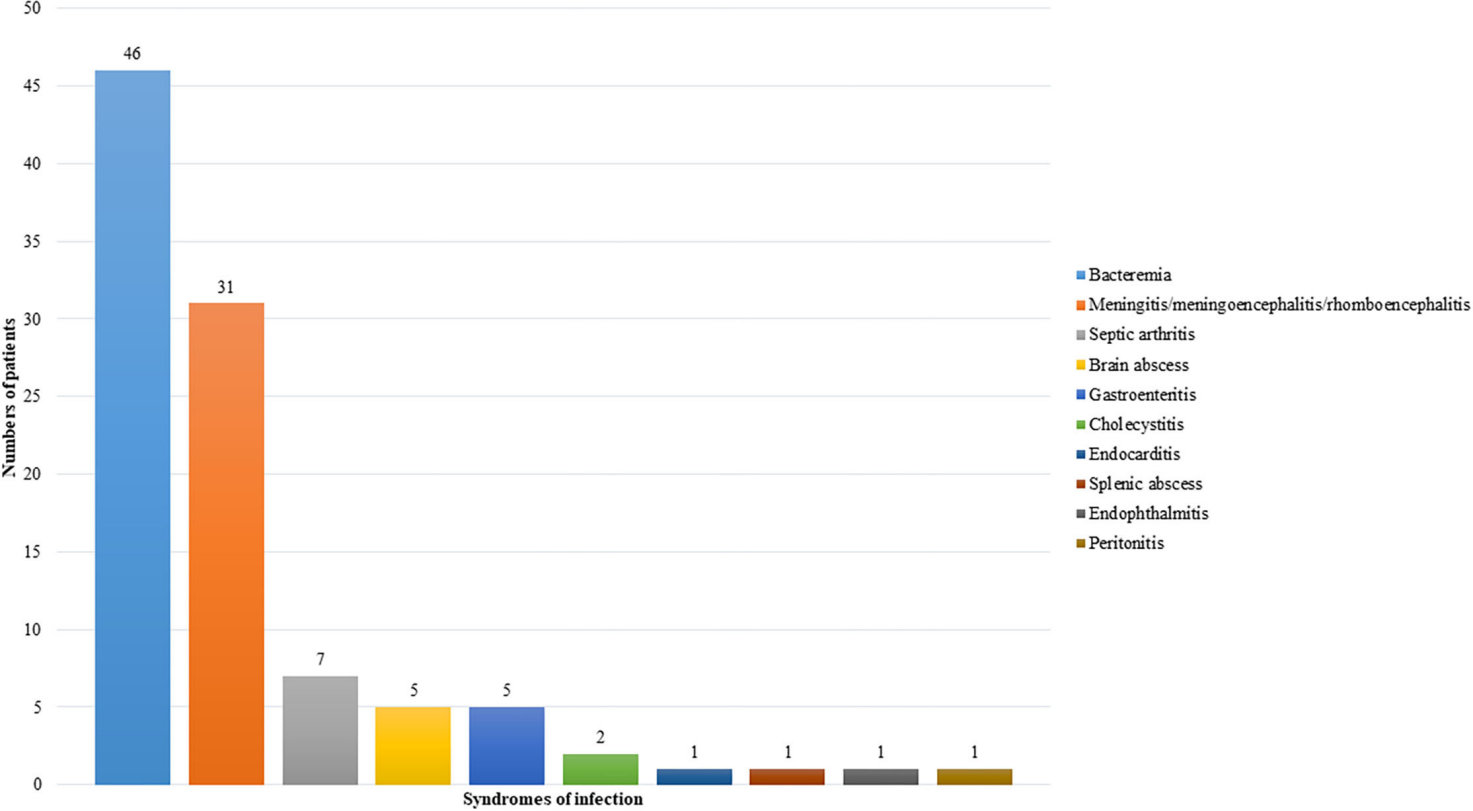
Types of listeria infection in patients on targeted therapy.

Targeted therapy is an important predisposing factor for listeriosis. The first patient reported to have listeria infection complicating targeted therapy was a patient with Crohn's disease on infliximab, an anti-TNFα monoclonal antibody (5). Since then, including the two cases in the present cohort, a total of 82 cases of listeriosis complicating targeted therapy have been reported in the scientific literature (5–37, 45–57). For 69 out of the 82 patients, gender, age, and clinical information were reported (5–11, 13–26, 28–32, 34–37, 45–57). The most common underlying diseases in these patients were autoimmune diseases (Figure 1A), whereas the commonest targeted therapy used was anti-TNFα monoclonal antibody (Figure 1B). In fact, the only case of listeriosis associated with inhibitors of the EGFR/HER2 pathway in the literature was a patient with metastatic breast cancer who received trastuzumab and lapatinib (47). The two patients in the present cohort who received gefitinib/osimertinib/afatinib and trastuzumab/lapatinib, respectively represent the second and third cases of patients with listeria infections related to inhibitors of the EGFR/HER2 pathway. For the listeria infection, the majority had bacteremia (Figure 2), in line with the present cohort where all five patients were blood culture positive. Central nervous system infection, present in one of our patients, was also commonly observed (Figure 2). Interestingly, around 80% of the listeria cases complicating targeted therapy were reported from the western world (Figure 1C). We speculate that this is at least partially due to the dietary habits and culinary traditions in these countries. It is important to note, but many clinicians may not be aware of, that listeria bacteria are resistant to cephalosporins, therefore in suspected cases ampicillin or penicillin should be added. As shown in the present cohort, two out of the five patients with listeriosis were on targeted therapy, which made it an important risk factor to consider. Therefore, listeriosis is an important differential diagnosis in patients on targeted therapy who present with sepsis and/or central nervous system infection and the use of antibiotic regimens that cover listeria is crucial for empirical treatment. Furthermore, avoidance of high-risk food items in these patients is important for the prevention of listeriosis.

The incidence of listeriosis could be underestimated. One of the most important causes of culture-negative infections is partially treated pyogenic bacterial infections. In the present cohort, although *L. monocytogenes* was isolated from the blood culture of case 5, it could not be recovered from the cerebrospinal fluid, despite the fact that there was increased white cell count and protein in the cerebrospinal fluid. Subsequently, the cerebrospinal fluid was sent for next-generation sequencing which confirmed the diagnosis of listeria meningitis. In 2020, we encountered a 26-year-old previously healthy woman who presented with recent onset of fever, severe headache and altered mental function. She had diarrhea for 4 days and has been treated with antibiotics. On admission, her temperature was 40.1°C. She also had neck rigidity, delirium and nystagmus. Examination of the cerebrospinal fluid revealed elevated white cell count with predominant neutrophils, increased protein and decreased glucose levels. Although Gram-smear revealed abundant Gram-positive rods, no bacteria could be isolated. All other microbiological investigations were negative. We speculate that this patient also had listeria meningoencephalitis, although it could not be confirmed microbiologically, as the patient had received antibiotics for a few days before lumbar puncture. She was treated as such accordingly and then recovered. These two cases illustrated that listeria infections could have been underestimated. A high index of suspicion is important and the use of next-generation sequencing would be useful for identifying more cases of partially treated listeriosis (40, 58).

## Data Availability Statement

The original contributions presented in the study are included in the article/supplementary material, further inquiries can be directed to the corresponding author/s.

## Ethics Statement

The studies involving human participants were reviewed and approved by the Institutional Review Board of The University of Hong Kong - Shenzhen Hospital. The patients/participants provided their written informed consent to participate in this study. Written informed consent was obtained from the individual(s) for the publication of any potentially identifiable images or data included in this article.

## Author Contributions

FX and PW conceptualized the study and wrote the manuscript. FX reviewed the clinical data. SKFL supervised the microbiological investigations. All authors have read and approved the final version of the manuscript.

## Funding

This study was partly supported by Sanming Project of Medicine in Shenzhen, China (SZSM201911014) and the seed fund of The University of Hong Kong-Shenzhen Hospital (HKUSZH201901036). The funders had no role in study design, data collection or analysis, decision to publish, or preparation of the manuscript.

## Conflict of Interest

The authors declare that the research was conducted in the absence of any commercial or financial relationships that could be construed as a potential conflict of interest.

## Publisher's Note

All claims expressed in this article are solely those of the authors and do not necessarily represent those of their affiliated organizations, or those of the publisher, the editors and the reviewers. Any product that may be evaluated in this article, or claim that may be made by its manufacturer, is not guaranteed or endorsed by the publisher.
